# Analysis the Link between Periodontal Diseases and Alzheimer’s Disease: A Systematic Review

**DOI:** 10.3390/ijerph18179312

**Published:** 2021-09-03

**Authors:** Leslie Borsa, Margaux Dubois, Guillaume Sacco, Laurence Lupi

**Affiliations:** 1 Faculté de Chirurgie Dentaire-Odontologie, Université Côte d’Azur, 06300 Nice, France; borsa.l@chu-nice.fr (M.D.); Laurence.LUPI@univ-cotedazur.fr (L.L.); 2 Pôle Odontologie, Centre Hospitalier Universitaire de Nice, Université Côte d’Azur, 06000 Nice, France; 3 UPR7354–Laboratoire Microbiologie Orale, Immunothérapie et Santé (Micoralis), Faculté de Chirurgie Dentaire-Odontologie, Université Côte d’Azur, 06300 Nice, France; 4 Clinique Gériatrique du Cerveau et du Mouvement, Centre Hospitalier Universitaire de Nice, Université Côte d’Azur, 06003 Nice, France; sacco.g@chu-nice.fr; 5 Université Côte d’Azur, CoBTeK, 06100 Nice, France; 6 Univ Angers, Université de Nantes, LPPL, SFR CONFLUENCES, 49000 Angers, France

**Keywords:** Alzheimer’s disease, periodontal disease, periodontitis, oral microbiota, *Porphyromonas gingivalis*

## Abstract

The hypothesis of an infectious connection from the oro-pharyngeal sphere to the brain underlines the interest in analyzing the link between periodontal disease and Alzheimer’s disease. The aim of this systematic review was to examine the link between Alzheimer’s disease and periodontal disease in patients aged 65 and over. Databases (PubMed (MEDLINE), the Cochrane Library, and Embase) were analyzed for relevant references up to 21 June 2021. The authors independently selected the studies and extracted the data. The quality of included studies was checked using the National Institutes of Health’s quality assessment tools. Five studies were included. The selected studies described in their results an increase in *F. nucleatum* in Alzheimer’s disease patients (adjusted *p* = 0.02), and its incidence was linked to *C. rectus* and *P. gingivalis* (adjusted HR = 1.22 (1.04–1.43), *p* = 0.012) as well as *A. naeslundii* (crude HR = 2.0 (1.1–3.8)). The presence of periodontitis at baseline was associated with a six-fold increase in the rate of cognitive decline over a 6-month follow-up period (ADAS-Cog mean change = 2.9 ± 6.6). The current review suggests an association between periodontal disease and Alzheimer’s disease. The treatment of periodontal disease could be a way to explore Alzheimer’s disease prevention.

## 1. Introduction

Around 770 microorganisms compose the human oral microbiota. This microbiota forms a biofilm or dental plaque on the surface of teeth. Its composition is dynamic and depends on the host environment. In a healthy state, with good dental hygiene, the composition of the microbiota remains stable [1]. This ecosystem is in a fragile balance to maintain oral health. In healthy conditions, Gram-positive bacteria dominate the composition of the dental plaque. However, because of different factors such as sugars, acids, or a lack of tooth brushing, the plaque composition could evolve into having a greater proportion of Gram-negative anaerobic bacteria [2]. This altered balance, also named oral dysbiosis, can lead to oral pathologies such as periodontal disease (PD). The inflammation, which starts in the gum area, is called gingivitis. When the gum inflammation persists and progresses, it creates the conditions for the development of periodontitis [3,4], which can spread to the alveolar bone, the cementum, and the periodontal ligament. It is then characterized by the formation of periodontal pockets, alveolar resorptions, and periodontal recessions. Periodontitis is a frequent, chronic, and inflammatory disease. Its etiology is mainly infectious, which affects the supporting tissues of the tooth. The pathogenicity of each species taken alone is relatively low, and cooperation between the bacteria is necessary. The periodontopathogenic bacteria *Porphyromonas gingivalis* (*Pg*), *Prevotella intermedia* (*Pi*), *Treponema denticola* (*Td*), *Tannerella forsythensis (Tf)*, *Campylobacter rectus* (*Cr*), *Aggregatibacter actinomycetemcomitans* (*Aa*), and *Fusobacterium nucleatum* (*Fn*) release inflammation mediators and toxins, which go into the bloodstream and participate in systemic inflammation. Consequently, chronic periodontitis can contribute to the development of various pathologies [5].

Alzheimer’s disease (AD) is the leading cause of neurocognitive disorders in the adult population, with nearly 47 million people affected worldwide. The estimated annual cost worldwide has exceeded 1000 billion US dollars [6], and except for severe neurodevelopmental disorders, disability related to neurocognitive disorders was the most significant compared to all other pathologies [7]. Despite progress made in the last 30 years in the understanding of neuropathological mechanisms, particularly concerning tau and beta-amyloid processes [8], the question of the etiopathogeny and risk factors of AD is still debated [9,10]. Among the risk factors studied, chronic inflammation and infectious pathologies constitute an interesting path of research [11]. In recent years, the hypothesis that PD is a risk factor for AD has been growing [12,13]. The oral cavity seems to represent a privileged observation space to study these issues because of the established link between oral health and more than fifty systemic diseases, including cardiovascular and pulmonary diseases, diabetes, and some forms of cancer [14,15,16]. The link between PD and AD could be explained by the spread of infectious or inflammatory agents migrating from the oral cavity to the brain [17,18,19,20]. According to Miklossy, J. and McGeer [21], a seven-fold-higher density of oral bacteria is found in the brain tissue of deceased AD patients compared to controls. The oral bacteria most found in the central nervous system and associated with cognitive impairment are spirochetes (Treponema) [22], *Pg* [23], *Fn*, *Pi*, *H. pylori*, and *B. burgdorferi*. In particular, *Pg*, the key pathogen of chronic periodontitis, is significantly identified in the brains of patients who have died from AD. Moreover, in mice models, an infection with *Pg* results in a colonization of the brain by this bacterium, and in an increased production of amyloid plaques within it [24].

The composition of the oral microbiota in AD patients and its association with the severity of the disease remain largely unknown. In the absence of an effective disease-modifying treatment for AD, a great deal of research is being carried out to prevent its onset and/or progression. It is therefore necessary to describe and understand the link between PD and AD. The questions are therefore these: “Is there a link between PD and AD?”, and if so, "What would be the nature of this link?”

## 2. Materials and Methods

### 2.1. Identification of Research Question

We carried out a systematic review of the scientific literature and reported the results according to the Preferred Reporting Items for Systematic Reviews and Meta-Analyses (PRISMA) guidelines [25] (supplementary material, Table S1). The study was registered in PROSPERO, registration number is CRD42021270806.

Research questions were defined to answer the research objectives: “Is there a link between PD and AD?” and “What would be the nature of this link?” The PICO question was “Are people aged 65 and over (P) who have PD (I) compared with those without PD (C) at increased risk for AD (O)?”

### 2.2. Goals

The primary goal was to determine if a link between PD and AD can be said to clinically exist based on periodontal clinical markers in people over 65 years of age.

The secondary goal was to determine the microbiological pattern (the presence of specific periodontal bacterial pathogens) in people over 65 years of age with AD.

### 2.3. Elligibility Criteria

The conditions for eligibility were specified before the onset of screening.

The inclusion criteria were as follows: (i) human studies in which participants over 65 years of age were at least a part of the study sample; (ii) the analysis of the link between PD and AD was one of the objectives of the study; (iii) the evaluation of the PD and/or the bacterial microbiota related to PD was clearly defined; (iv) the diagnosis criteria for AD diagnosis were clearly defined; (v) clinical studies, longitudinal studies (cohort or case–control; retrospective and/or prospective), and transversal studies that evoked the link between periodontal disease and Alzheimer’s disease; (vi) studies published between 2010 and 21 June 2021; and (vii) studies written in English.

Studies interested in other syndromes (e.g., Down syndrome or neurocognitive disorders not related to AD) and experimental studies on animals or on human tissues were excluded.

### 2.4. Information Sources and Search Strategy

The bibliographical research was performed through the following electronic databases: PubMed (MEDLINE)/the Cochrane database (Cochrane Library)/Embase.

The keywords used were “Alzheimer”, “Alzheimer disease”, “periodontal disease”, “periodontitis”, “calculi”, “calculus”, and “dental plaque”. Keywords were mixed using Boolean operators, and MeSH terms (for MEDLINE and Cochrane) or Emtree (for Embase) were used depending on the database. For example, the search strategy used for PubMed (MEDLINE) database was (“Alzheimer’s disease” (MeSH) or “Alzheimer”) and (“Periodontal disease” (MeSH) or “Periodontitis” (MeSH) or “dental plaque” (MeSH) or “calculi” (MeSH) or “calculus”).

### 2.5. Selection Process

Two authors manually extracted data independently. First, duplicates were removed. Then, articles of interest were selected based on their titles and abstracts. The final selection of the articles to be included was based on reading the full texts, according to the PICO characteristics, as well as inclusion and exclusion criteria. Reasons for exclusion were documented at each stage. Any discrepancy regarding references, selection criteria of the subjects, sample characteristics (number and age of participants as well as sample size calculation), diagnosis criteria of AD, diagnosis criteria of PD, outcomes and relevant data, follow-up duration, and results were solved by discussion between the authors.

### 2.6. Data Collection Process

For each eligible study, the following data were extracted by two members of the research team: first author, year of publication, study type, protocol, total number of patients, and covariates studied. The average age and the number of females were also extracted.

### 2.7. Risk of Bias

The National Institutes of Health’s (NIH) study quality assessment tools for observational cohort and cross-sectional studies in addition to those of case–control studies were used to assess the quality of each eligible study [26]. In the case of any discrepancy, a consensus was reached after discussion and re-evaluation between two members of the research team, and, if necessary, the opinion of a third member of the review team was requested.

## 3. Results

**Figure 1:**
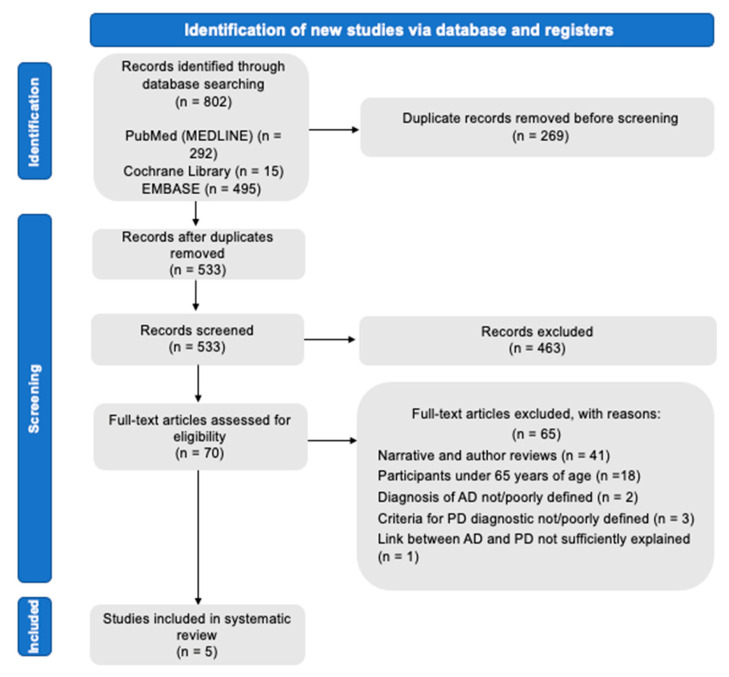
PRISMA flowchart diagram.

A total of 802 articles were identified. After elimination of duplicates, 533 articles were retained. We finally obtained 70 articles to read fully. Among them, five articles were finally included in the qualitative analysis (Figure 1).

All the selected articles analyzed the link between PD and AD: three case–control studies [27,28,29], one observational cross-sectional study [30], and one cohort study [31] were analyzed.

### 3.1. Characteristics of the Included Studies

The characteristics of the included studies are presented in the supplementary material, Table S2.

Only five studies met the inclusion criteria, and they provided data from populations in four countries: Italy [27], USA [28,30], UK [31], and Finland [29]. The number of participants ranged from 59 to 3251. The follow-up period for the only cohort study was 6 months [31].

Three studies [27,30,31] considered both clinical and bacterial criteria to define PD, one study [28] only considered bacterial criteria, and one study [29] only considered clinical criteria.

The tests used for AD diagnosis were the Mini-Mental State Examination (MMSE) test [27,28,31], the Diagnostic and Statistical Manual of Mental Disorders, 4th Edition (DSM-IV) [28,29], the Diagnostic and Statistical Manual of Mental Disorders, 4th Edition, Text Revision (DSM-IV-TR) [27], the Alzheimer’s Disease Assessment Scale–Cognitive Subscale (ADAS-Cog) [31], and the National Institute of Neurological and Communicative Disorders and Stroke and the Alzheimer’s Disease and Related Disorders Association (NINCDS-ADRDA) criteria [28,31].

The results of the association between AD and periodontal indicators are presented in the supplementary material, Table S3.

### 3.2. Bias and Quality of Included Studies

**Figure 2:**
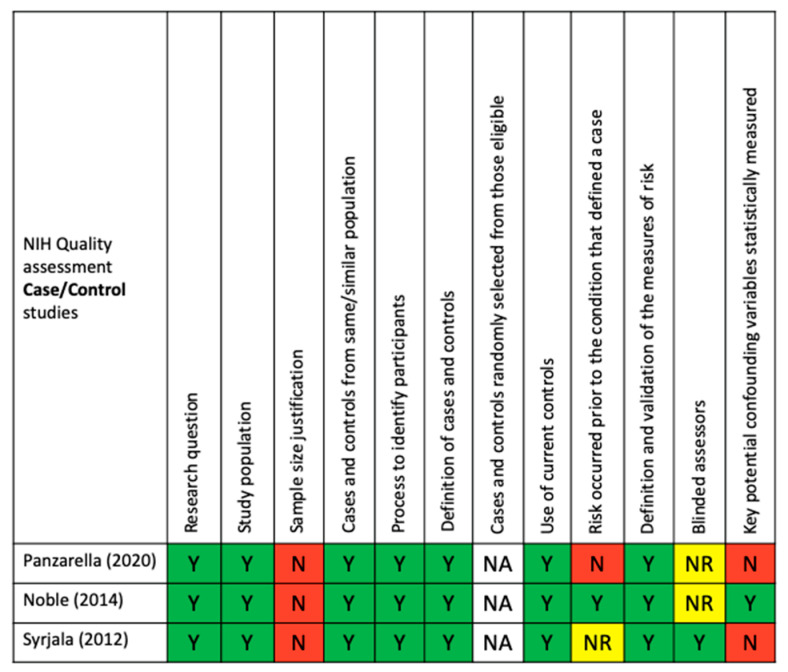
NIH quality assessment of case–control studies: Y, yes; N, no; NA, not applicable; and NR, not reported.

**Figure 3:**
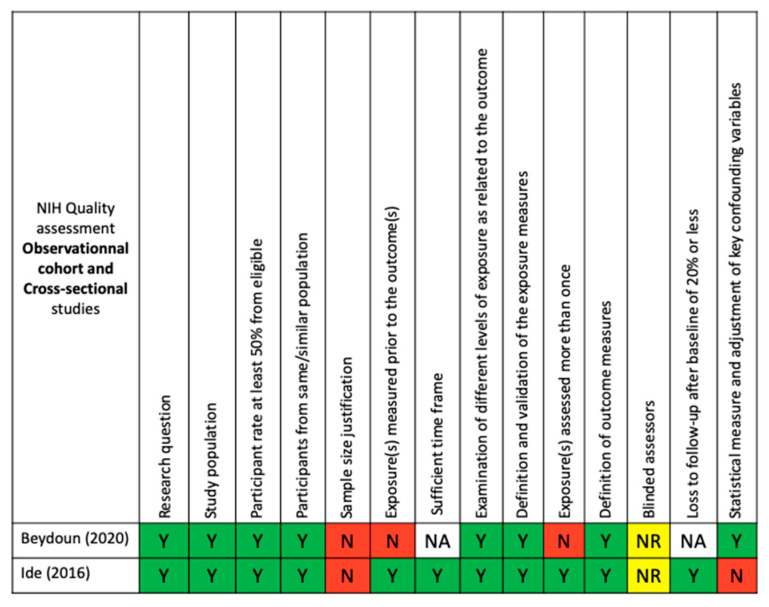
NIH quality assessment for observational cohort and cross-sectional studies: Y, yes; N, no; NA, not applicable; and NR, not reported.

Regarding bias, the quality of included studies was evaluated by the criteria of the NIH’s study quality assessment tools for observational cohort and cross-sectional studies in addition to those of case–control studies. Based on these criteria, two out of the five studies were qualified as good quality [28,29], while the remaining three studies [27,30,31] were rated as fair quality. The quality assessment tools for each selected study are presented in Figure 2 and Figure 3.

### 3.3. Association between AD and Periodontal Bacterial Pathogens

Four studies examined the association between periodontal bacterial pathogens and AD [27,28,30,31].

One study found a significantly higher *Fn* load in AD than in controls and a higher bacterial load of *Td* in persons with amnestic mild cognitive impairment than in AD [27]. Another study provided evidence of an association between periodontal pathogens and AD, which was even stronger for older adults [30]. One study did not find a significant relationship between the serum levels of anti-*Pg* antibodies and the rates of cognitive decline, but showed evidence of a relative increase in pro-inflammatory status and a decrease in anti-inflammatory status over a 6-month follow-up period in participants with AD and PD [31]. Another study showed that participants with elevated *A. naeslundii* (*An*) serum immunoglobulin G (IgG) had a consistently higher risk for incident AD, whereas high antibody levels to *E. nodatum* (*En*) were significantly associated with a decreased risk of incident AD [28].

### 3.4. Association between Alzheimer’s Disease and Clinical Periodontal Markers

Four studies examined the association between clinical periodontal markers and AD. One study estimated the diagnosis of AD as predictive of tooth loss, especially in subjects aged 80 years or older [27]. Another study showed that the presence of PD in AD was associated with a marked increase in cognitive decline over a 6-month follow-up period, independent of the basic cognitive state [31]. One study showed that persons with AD and persons with other types of major neurocognitive disorder (NCD) had an increased likelihood of having teeth with deep periodontal pockets compared to non-demented persons [29]. Two studies evoked the fact that the treatment of PD could be a way to explore AD prevention or treatment [30,31].

## 4. Discussion

### 4.1. Link between Alzheimer’s Disease and Periodontal Disease

This systematic review including five studies provides evidence of a relationship between AD and PD. However, these pieces of evidence on the relationship between PD and AD need to be further investigated.

In the studies analyzed, the diagnosis of PD was based on clinical criteria [29], microbiological criteria [28], or both types of criteria [27,30,31]. The choice to determine the PD status through the clinical diagnosis of PD may be relevant if it is based on a complete periodontal charting by registering several parameters (probing pocket depth (PPD), clinical attachment level (CAL), bleeding on probing (BOP), and plaque index (PI)) [32]. While the PPD was evaluated in all studies including a clinical diagnosis of PD [27,29,30,31], the clinical attachment level (CAL) was measured in only one study [30], the BOP was listed in one study [27], and the PI was registered in two studies [29,31]. The choice to apprehend PD status through bacteriological considerations may also be relevant, since it is known that certain bacteria and/or bacterial complexes are unfavorably associated with a state of PD [12,33,34]. Four studies analyzed the microbial status of participants [27,28,30,31]. The evaluation of the PD status through clinical and microbiological data was only done in three studies [27,30,31]. One study [28] used serological tests to select moderate to severe stages of PD. Ide et al. [31] refer to periodontitis by explaining that it was considered according to its moderate and severe forms, but without detailing them in the different tables or in the article itself. Indeed, only the presence or absence of periodontitis seems to be considered in the end. However, this publication also concludes that there was no clear relationship between the degree of PD and the severity of AD.

The initial examination leading to a probable diagnosis of AD, according to the DSM V, generally includes different aspects such as a cognitive assessment (including a global evaluation, usually with the MMSE), a functional assessment (IADL or ADL scales), a behavioral assessment, a clinical examination, and para-clinical examinations (biological examinations and magnetic resonance imaging (MRI)) [35], since only a lumbar puncture can establish a diagnosis of high probability, which is not required as a routine examination for this type of diagnosis.

If the MMSE test [36,37] is used on a recurrent basis [27,28,31], there are differences between the studies, as not all of them use the same reference systems to establish a diagnosis of AD. Two studies [28,29] used the DSM-IV criteria [38], and one other [27] used the DSM-IV-TR [39]; a criterion of the American Psychiatric Association and included in the Diagnostic and Statistical Manual of Mental Disorders in 1994 [38] and 2000 [39], one study [31] used the ADAS-Cog [40], while others [28,31] used the criteria of the NINCDS-ADRDA [41]. Some studies have used a combination of several tests [27,28,31]. One study, using database results [30], selected patients based on their membership of the category of people with AD according to the codes of the International Classification of Diseases (ICD), version nine, for the diagnosis of AD, and the ICD, version 10, code G30, for the underlying cause of death [42,43]. Three studies used DSM-IV [27,28,29] in combination [27,28], or not [29], with other diagnostic tests. Only one study [27] refers to the use of MRI in the diagnosis of AD.

One study [29] considered the stage of major NCD, but did not associate it with a specific type of major NCD, and moreover did not really use it to assess the presence of PD, but to assess oral hygiene.

We wished to study non-genetic forms, and because genetic aetiologias are considered rare after this age [44], we chose to include in our review only studies targeting people over 65, or to study only this age group when the study included several, for more homogeneity. We also wished to focus our research on the clinical aspect of the link/association between the two diseases and therefore chose to exclude studies of a genetic type or that were carried out in vitro.

All the studies analyzed chose to include covariates such as age [27,28,29,30,31], sex [27,28,29,30,31], tobacco consumption [27,28,29,30,31], alcohol [27,30] or drug consumption [30], education [28,29,30], housing [29,30], income [30], marital status [29,30], ethnicity [28,30], sports activity [30], nutrition [30], and medical factors, such as obesity [27,30], hypertension [27,28], diabetes [27,28], cholesterol [27,28], a history of stroke [28], APoE4 carrier status [27,28], heart disease [27,28], allostatic load [30], and basic cognitive status [31], which can be potential confounding factors as some of these are common risk factors for PD and major NCD.

In AD, poor dental health, particularly the presence of PD, is associated with a marked increase in cognitive decline over a 6-month follow-up period [31]. However, this does not appear to be a relationship between low tooth counts, a possible indicator of past PD, and cognitive decline, suggesting that chronic active periodontitis is more important in driving cognitive decline once AD is established, and suggesting a direct relationship between PD and cognitive decline. However, no clear relationship has been shown between degree of PD and severity of major NCD [31]. Indeed, patients with AD are more likely to have poor oral hygiene and poorer oral health related to PD compared to people without AD [27,29]. Treatment of periodontitis may therefore be part of the care strategy in AD [31].

Whereas Ide et al. [31] showed that in AD patients the presence of PD is associated with a marked increase in cognitive decline, and reported evidence of a relative increase in pro-inflammatory status and a decrease in anti-inflammatory status over a 6-month follow-up period in participants with PD, they did not show a significant relationship between *Pg* antibody serum levels and the rate of cognitive decline, while others have shown a link between AD and certain periodontal pathogens [27,28,30]. Thus, the bacterial load of *Fn* appears significantly higher in AD [27]. The incidence of AD is increased in participants with high levels of IGg antibodies to *An*, and this appears to be even stronger when high levels of *En* are included in the model [28]. According to Beydoun et al. [30], *Pg*, *Pi*, *P. nigrescens (Pn)*, *Fn*, *Cr*, *S. intermedius (Si)*, *C. ochracea (Co)*, and *P. melaninogenica (Pm)* could be related to increased AD mortality risk above 65 years of age, while the reverse could be true for Aa. *S. oralis* (*So*) was directly related to AD mortality risk among men. *Pg* and *Cr* were linked to an increased risk for incidence of AD over 65 years of age. *So* increased the risk of major NCD (all causes) in men, while *E. corrodens (Ec)* increased the risk of major NCD (all causes) in women. The reverse was true for *Si*, which appeared to be marginally and inversely associated with the risk of AD incidence in women [30]. This provides evidence of an association between periodontal pathogens and AD, which is even stronger for older adults. There is epidemiological evidence suggesting that eradication of *Pg*, among others, could be an effective way to delay the onset of AD, pending randomized clinical trials [30].

Further multimodal exploration of the role of periodontal infections, other oral health markers, and systemic host response may elucidate a potential new causal pathway for cognitive impairment in the elderly [28].

### 4.2. Limitation of Selected Studies

The NIH’s study quality assessment tools were used to assess the quality of each eligible study (Figure 2 and Figure 3).

One of the major limitations of all the studies found for this systematic review was the diagnosis criteria used to define AD patients. Indeed, criteria used in these studies allowed for the diagnosis of neurocognitive disorders associated with Alzheimer’s syndrome but, without a CSF sample, the proteinopathy related to AD could not be established. Thus, linking PD and AD on this basis could be controversial. The only conclusion possible is the association between PD and NCD. To confirm the hypotheses of an association between PD and AD, and to explore the possible physiopathological process underlying these two pathologies, future studies should include only well-phenotyping AD patients with a confirmed amyloïdopathy, according to the last National Institute on Aging and Alzheimer’s Association Research Framework [45].

It is also worth noting the multiplicity of criteria used to define PD in the selected publications. Moreover, three studies (Beydoun et al. [30], Panzarella et al. [27], and Noble et al. [28]) used existing databases. Thus, the investigators did not collect the data themselves, and this could have led to selection biases such as misclassification of participants [27], underdiagnosis of AD, or underestimation of the severity of periodontal disease [30], due to the lack of specific data [28]. Both aspects make a strict comparison between the different results difficult. The use of more stringent and standardized methods seems to be necessary to obtain a more reliable diagnosis. For PD, a systematic integration of a clinical and a microbiological component may be needed.

Calibration of dental examiners prior to the data collection period has been described as weak by Syrjälä et al. [29], with furthermore a lack of assessment of the reliability of oral examinations between and within examiners [29]. The database studies [27,28,30] did not mention this problem; nevertheless, it may exist, leading to bias in the selection of those with and without PD. The other study [31] which did not use a database evokes a collection of periodontal clinical parameters carried out by a research dental hygienist, which assumes a minimum of calibration, but does not state this.

It should be noted that some of the variables used in the selected studies correspond to common risk factors for both periodontitis and major NCD, and may induce errors in the interpretation of the relationship between these two processes. The measurable socio-economic covariates provide a limited range of socio-economic status over the course of life and could lead to residual confusion. However, the influence of socio-economic differences has been reduced by case matching and controls on race and ethnicity. Adjustment for chronological age alone may not capture residual confounders, due to age-related diseases that are variably distributed among people of similar age [28].

While some studies [28,29,31] refer to different stages of major NCD or different degrees of PD, none clearly use these data in their analysis or in a comparative manner between AD and PD, and only the presence or absence of PD and AD are actually used. For one study [31], the presence of PD means a moderate or severe stage, but it does not distinguish between the two. It would therefore have been interesting to be able to classify the diseases according to their stage of disease in order to make clear comparisons between the severity of major NCD and the severity of PD, especially as the suggested mechanisms linking PD or periodontal pathogens to cognitive impairment and major NCD are still speculative [30]. It is possible that participants with a faster rate of cognitive decline may become more susceptible to periodontitis by an unknown mechanism independent of the degree of cognitive impairment, or that periodontitis reflects a cofounding factor such as an altered inflammatory or immune response, which is also a factor in the progression of AD. On the other hand, PD may be a direct factor in disease progression [31].

Furthermore, the sample sizes were not uniform and homogeneous between the different studies analyzed. With the exception of the Beydoun and al. study [30], a cross-sectional observational study that used a large sample of 6650 subjects, including 3251 people aged over 65 years, the sample sizes of the studies were always relatively small, which gives the studies little power and potentially increases the likelihood of false associations and lack of significance. Three studies were case–control studies and used small sample sizes. Panzarella et al. focused on 40 major NCD cases, including 20 AD cases and 20 controls [27]. The study by Noble et al. analyzed 110 cases and 109 controls [28]. The study by Syrjälä et al. observed 76 subjects with major NCD among them; 49 were AD cases and 278 were controls [29], thus leading to a lack of homogeneity between the number of cases and controls. The study by Ide et al. was a cohort study and included a small sample of 59 people with AD [31]. This sample and design variability between the different studies makes it difficult to compare results. Future studies should therefore reduce heterogeneity within and between groups [27].

### 4.3. Periodontal Bacteria: Towards a Better Understanding of the Link

Several studies have shown that the clinical parameters for diagnosing periodontal disease and assessing its severity, such as PPD, CAL or BOP, are significantly increased in patients with AD [46,47,48]. Indeed, according to Gil de Montoya’s study [48], the risk of cognitive disorders is three times higher for patients with severe PD compared to those who do not have it or who have moderate PD (OR = 3.04) (1.69–5.46). A pocket depth of more than 6 mm, which corresponds to severe periodontitis, would thus be associated with a 15 times greater risk of developing AD [49].

Furthermore, there appears to be a link between disease severity and cognitive decline [50], as patients with severe stage III and IV periodontitis have the lowest MMSE and CDT scores. However, this is refuted by other authors [49].

In addition, other authors stress the importance of the duration of periodontal disease. Patients with chronic periodontitis are reported to be at greater risk of developing AD [30,51]. Indeed, even if the study by Chen et al. [46] shows no statistically significant relationship between patients with periodontal disease and controls, a difference appears after 10 years of exposure to PD (OR 1.71, 95% CI (1.15–2.5”), and *p* = 0.0077); patients exposed for a long time would then have 1.7 times more risk of developing AD than those with a healthy periodontium.

This may explain why some studies do not show a link between the two diseases, as it seems that cause precedes effect [52].

Genetics does not appear to be a causal factor in the link between the two diseases [53].

While there is currently no clinical evidence with which to describe a direct link between PD and AD, several studies propose indirect links. The two main hypotheses are inflammation and the bacteria involved. Periodontal pathogenic bacteria cause an inflammatory defense reaction in the host, leading to the production of inflammatory molecules such as IL-1B, IL-6, IL-8, and TNF. In advanced PD, the inflammatory cytokines are able to reach the central nervous system via the bloodstream, which is believed to influence the progression of AD [23,24,28,54,55,56,57].

Research into bacteria involved in the onset and/or progression of AD has highlighted many risk associations. *Pg*, a highly periodontal pathogenic bacteria, was the first to be incriminated. Its presence is thought to be linked to an increase in cognitive decline in patients and its presence in saliva is statistically linked to lower MMSE (*p* < 0.05) and CDT (*p* = 0.056) scores [50].

Other periodontopathogenic bacteria have been the subject of studies aimed at gaining a better understanding of their link with the onset and/or progression of Alzheimer’s disease. The study by Beydoun et al. [58] reveals the synergy of many periodontopathogenic bacteria (*Pi*, *An*, *Pn*, *Pm*, *Pg*, *Td*, and *Tf*) with *Helicobacter pylori* and their impact on AD.

The role of *Pg* in the etiopathogenesis of AD has also been highlighted by Poole et al. [54] The bacterium, anti-*Pg* antibodies, and its lipopolysaccharides have been found in the brain and cerebrospinal fluid of patients with AD [24,54]. This means that *Pg* is able to bypass the immune system, reach the brain, and cross the brain barrier.

Recent study has proven the ability of periodontopathogenic bacteria to hijack the immune system [59]. They alter the function of macrophages and neutrophils. For example, during bacteremia caused by chewing, brushing teeth, or during treatment, they enter the brain via the bloodstream. To increase its virulence, *Pg* has the ability to secrete vesicles filled with proteases called gingipains. These have the function of breaking down cytokines to reduce inflammation. They use the tau protein present in the brain as a substrate. Its modification transforms the molecule into a neurotoxic product that contributes to the progression of AD. Gingipains can also be absorbed by neural cells to be detoxified. They will be transported from cell to cell throughout the neural system, spreading the infection that causes cognitive decline [59,60].

It should be noted that the improvement in the PI favorably modifies the oral microbiological composition [61,62,63] and could make it possible to reduce the incidence of pathogens in neuronal pathology. This hypothesis is a feasible means of attempting to reduce the incidence of major neurocognitive disorders in the elderly, and would justify oral health monitoring as well as more systematic dental management of the elderly.

Few studies currently have a high level of evidence on the link between AD and PD [64,65]. This link seems to be mainly described in animals, and when there are clinical studies in humans many of them only make a statement about periodontal status and AD without really analyzing it [46,49,52,65,66].

Moreover, studies often have small numbers of staff [23,29,49,50,52,66,67]. Larger, multi-center studies should be undertaken in this way.

Most studies analyze the link only at a point in the patient’s life [23,28,29,46,47,49,50,53,58,67], but many authors suggest that periodontal diseases influence AD long before the first symptoms appear.

Finally, as many risk factors have been established with AD, risks of confounding bias are often described by the authors [30,46,51,58].

## 5. Conclusions

The literature currently seems to converge towards the affirmation of a link between periodontal diseases and Alzheimer’s disease. Indeed, AD patients seem to have a lower level of oral hygiene and a higher risk of PD. In this context, periodontopathogenic bacteria have been found in AD patients, either associated with a higher risk of AD incidence or mortality, or inversely associated with these risks. Among them, *Pg*, *Fn*, *An*, and *Aa* seem to be the key bacteria. Nevertheless, further studies are still needed to better understand the origin and mechanisms of this interaction, as there are not yet enough studies with a high level of evidence on the subject. Thus, new studies that are better constructed and more rigorous in the diagnostic criteria of the pathologies studied are essential to try to better understand the association between AD and PD.

It seems fundamental to focus on this association, as a better understanding of it will allow the implementation of effective prevention and even treatment measures. Thus, the treatment of PD could represent a lever in a global strategy for the management of the prevalent and disabling disease that is AD.

## Data Availability

No new data were generated.

## Supplementary Materials

The following are available online at https://www.mdpi.com/article/10.3390/ijerph18179312/s1, Table S1. PRISMA 2020 checklist, Table S2. Characteristics of the included studies, and Table S3. Main results of the included studies.
