# Supplementary material for: Analysis the Link between Periodontal Diseases and Alzheimer’s Disease: A Systematic Review
Source: Int J Environ Res Public Health. 2021 Sep 3;18(17):9312. doi: 10.3390/ijerph18179312 (PMC8430572; doi:10.3390/ijerph18179312)
Supplement: Supplementary file 1 [file ijerph-18-09312-s001.zip › ijerph-1330580-supplementary.pdf]

# Supplementary Materials

**Table S1. PRISMA 2020 Checklist.**

| Section and Topic | Item # | Checklist item | Location where item is reported |
|---|---|---|---|
| **TITLE** | | | |
| Title | 1 | Identify the report as a systematic review. | 1 |
| **ABSTRACT** | | | |
| Abstract | 2 | See the PRISMA 2020 for Abstracts checklist. | 1 |
| **INTRODUCTION** | | | |
| Rationale | 3 | Describe the rationale for the review in the context of existing knowledge. | 2 |
| Objectives | 4 | Provide an explicit statement of the objective(s) or question(s) the review addresses. | 2 |
| **METHODS** | | | |
| Eligibility criteria | 5 | Specify the inclusion and exclusion criteria for the review and how studies were grouped for the syntheses. | 2-3 |
| Information sources | 6 | Specify all databases, registers, websites, organisations, reference lists and other sources searched or consulted to identify studies. Specify the date when each source was last searched or consulted. | 3 |
| Search strategy | 7 | Present the full search strategies for all databases, registers and websites, including any filters and limits used. | 3 |
| Selection process | 8 | Specify the methods used to decide whether a study met the inclusion criteria of the review, including how many reviewers screened each record and each report retrieved, whether they worked independently, and if applicable, details of automation tools used in the process. | 3 |
| Data collection process | 9 | Specify the methods used to collect data from reports, including how many reviewers collected data from each report, whether they worked independently, any processes for obtaining or confirming data from study investigators, and if applicable, details of automation tools used in the process. | 3 |
| Data items | 10a | List and define all outcomes for which data were sought. Specify whether all results that were compatible with each outcome domain in each study were sought (e.g. for all measures, time points, analyses), and if not, the methods used to decide which results to collect. | 3 |
| | 10b | List and define all other variables for which data were sought (e.g. participant and intervention characteristics, funding sources). Describe any assumptions made about any missing or unclear information. | 3 |
| Study risk of bias assessment | 11 | Specify the methods used to assess risk of bias in the included studies, including details of the tool(s) used, how many reviewers assessed each study and whether they worked independently, and if applicable, details of automation tools used in the process. | 3 |
| Effect measures | 12 | Specify for each outcome the effect measure(s) (e.g. risk ratio, mean difference) used in the synthesis or presentation of results. | Table II |
| Synthesis methods | 13a | Describe the processes used to decide which studies were eligible for each synthesis (e.g. tabulating the study intervention characteristics and comparing against the planned groups for each synthesis (item #5)). | 3 |
| | 13b | Describe any methods required to prepare the data for presentation or synthesis, such as handling of missing summary statistics, or data conversions. | NA |
| | 13c | Describe any methods used to tabulate or visually display results of individual studies and syntheses. | NA |
| | 13d | Describe any methods used to synthesize results and provide a rationale for the choice(s). If meta-analysis was performed, describe the model(s), method(s) to identify the presence and extent of statistical heterogeneity, and software package(s) used. | NA |
| | 13e | Describe any methods used to explore possible causes of heterogeneity among study results (e.g. subgroup analysis, meta-regression). | NA |
| | 13f | Describe any sensitivity analyses conducted to assess robustness of the synthesized results. | 3 |
| Reporting bias assessment | 14 | Describe any methods used to assess risk of bias due to missing results in a synthesis (arising from reporting biases). | 3 |

| Section and Topic | Item # | Checklist item | Location where item is reported |
|---|---|---|---|
| Certainty assessment | 15 | Describe any methods used to assess certainty (or confidence) in the body of evidence for an outcome. | 3 |
| **RESULTS** | | | |
| Study selection | 16a | Describe the results of the search and selection process, from the number of records identified in the search to the number of studies included in the review, ideally using a flow diagram. | 4 |
| | 16b | Cite studies that might appear to meet the inclusion criteria, but which were excluded, and explain why they were excluded. | NA |
| Study characteristics | 17 | Cite each included study and present its characteristics. | 4 |
| Risk of bias in studies | 18 | Present assessments of risk of bias for each included study. | 5-6 |
| Results of individual studies | 19 | For all outcomes, present, for each study: (a) summary statistics for each group (where appropriate) and (b) an effect estimate and its precision (e.g. confidence/credible interval), ideally using structured tables or plots. | Tables |
| Results of syntheses | 20a | For each synthesis, briefly summarise the characteristics and risk of bias among contributing studies. | Tables |
| | 20b | Present results of all statistical syntheses conducted. If meta-analysis was done, present for each the summary estimate and its precision (e.g. confidence/credible interval) and measures of statistical heterogeneity. If comparing groups, describe the direction of the effect. | NA |
| | 20c | Present results of all investigations of possible causes of heterogeneity among study results. | 5-6 |
| | 20d | Present results of all sensitivity analyses conducted to assess the robustness of the synthesized results. | Figures 2 and 3 |
| Reporting biases | 21 | Present assessments of risk of bias due to missing results (arising from reporting biases) for each synthesis assessed. | 4-5 |
| Certainty of evidence | 22 | Present assessments of certainty (or confidence) in the body of evidence for each outcome assessed. | Table II |
| **DISCUSSION** | | | |
| Discussion | 23a | Provide a general interpretation of the results in the context of other evidence. | 9-10-11 |
| | 23b | Discuss any limitations of the evidence included in the review. | 8-9 |
| | 23c | Discuss any limitations of the review processes used. | 7-8 |
| | 23d | Discuss implications of the results for practice, policy, and future research. | 7-8-9-10 |
| **OTHER INFORMATION** | | | |
| Registration and protocol | 24a | Provide registration information for the review, including register name and registration number, or state that the review was not registered. | 2 |
| | 24b | Indicate where the review protocol can be accessed, or state that a protocol was not prepared. | NA |
| | 24c | Describe and explain any amendments to information provided at registration or in the protocol. | 2 |
| Support | 25 | Describe sources of financial or non-financial support for the review, and the role of the funders or sponsors in the review. | 11 |
| Competing interests | 26 | Declare any competing interests of review authors. | 11 |
| Availability of data, code and other materials | 27 | Report which of the following are publicly available and where they can be found: template data collection forms; data extracted from included studies; data used for all analyses; analytic code; any other materials used in the review. | 11 |

NA, Not Applicable

**Table S2.** Characteristics of the included studies

| Author and year | Type of study | Protocol | Characteristics of participants | Diagnosis method of AD | Diagnosis criteria of PD | Covariates studied |
|---|---|---|---|---|---|---|
| **Panzarella et al. 2020, J Alzheimers Dis**<br><br>**(27)** | Case control study | **Data** from the "Zabut Aging Project" (Italy) :<br><br>20 AD / 20 aMCI / 20 controls | Women = 55 %<br><br>Men = 45%<br><br>Mean age = 80 ± 8,68 yo, no significative differences between groups | Tests used:<br><br>MMSE, DSM IV-TR, MRI | **Bacterial criteria:** bacterial load for *Aa, Fn, Pg, Pi, Td, Tf*<br><br>**Periodontal criteria:**<br><br>-CPI = BoP, dental calculus, PPD<br><br>-PSR = mobility, muco-gingival damage, recessions >3.5 mm<br><br>-Abscess, halitosis, pain | Age, sex, tobacco, alcohol, level of education, obesity (body mass index = BMI), hypertension, diabetes, cholesterol, heart disease, DMFT, APoE Allele, hs-CRP |
| **Noble et al. 2014, Plos One**<br><br>**(28)** | Case control study | **Data** from the "multiethnic elderly community population" Manhattan (USA)<br><br>- 110 patients with AD when phlebotomy and follow-ups<br><br>- 109 controls: no AD/ major NCD at last follow-up | Control women = 73 (67%)<br><br>Case = 75 (68.2%)<br><br>Mean age of controls = 72 yo (s.d. 6.9)<br><br>Mean age of cases = 79 yo (s.d. = 4.6) ($p<0.001$) | Data from the WHICAP study<br><br>NINCDS-ADRDA DSM-IV<br><br>Tests used:<br><br>neuropsychological tests, ,MMSE | **Bacterial criteria:** Serum IgG antibody level against *Pg, Tf, Aa, Td, C, En, An* with definition of a threshold value | Age, gender, ethnicity, tobacco, grade level, ApoE e4 allele presence, vascular risk, hypertension, diabetes, diabetes, hyperlipidemia, stroke, heart disease |
| **Syrjälä et al. 2012, Gerontology (29)** | Case control study | Population over 75 yo Kuopio (Finland) N = 354<br><br>- 278 Controls<br><br>- 49 Cases with AD<br><br>- 16 Cases with VD<br><br>- 11 Cases with other types of major NCD | Women = 71.5%<br><br>- Control women = 71.2%<br><br>- AD women = 83.7%<br><br>- VD women = 56.2%<br><br>- Other types = 45.5%<br><br>Mean age = 82.0 yo (4.9) | Test used:<br><br>DSM-IV | **Periodontal criteria:** PI, PPD, number of teeth | Age, gender<br><br>level of education<br><br>tobacco, place of residence, marital status |

| | | | | | | |
|---|---|---|---|---|---|---|
| | | | Mean age without major NCD = 81.4 yo (4.6)<br><br>Mean age with AD = 84.8 yo (5.6)<br><br>Mean age with other types of dementia = 85.3 yo (4.8) | | | |
| **Beydoun MA. et al. 2020, J Alzheimers Dis**<br><br>**(30)** | Cross-sectional observational study | **Data** from NHANES III (USA): patients over 45 yo (n = 9787)<br><br>in which N = 3251 over 65 yo with complete medical data and known mortality | % of men = 41.4 +/- 1.12<br><br>Mean age = 73.6 +/- 0.24 yo | AD diagnosed with ICD-9 code 331.0<br><br>Underlying cause of death is AD with ICD-10 code G30 | **Bacterial criteria:** Complete serum IgG data against at least 1 of the 19 following periodontal bacteria*: Aa, Pg, Tf, Td, Cr, En, Pi, Pn, Pm, Fn, Mm, Sn, Ec, Co, Si, So, Sm, Vp, An*<br><br>**Periodontal Criteria:** CAL, PPD | Age, ethnicity, alcohol, drugs, tobacco, co-morbidity index, allostatic load, educational attainment, income, marital status, nutritional factors and markers, self-rated health, place of residence, weight, sport activity, sex, size of dwelling, self-rated health |
| **Ide et al. 2016, Plos One**<br><br>**(31)** | Longitudinal prospective study<br><br>=<br><br>observational cohort study | Recruitement for clinical referral to memory assessment services (Southampton, UK): non-smokers, mild to moderate AD, at least 10 teeth, no periodontal care in the last 6 months: N = 59<br><br>Re evaluation N +6 months = 52 | % of men = 30 (51%)<br><br>Mean age = 77,7 yo (s.d. 8,6) | AD diagnosed with the NINCDS-ADRDA criteria<br><br>Tests used:<br><br>cognitive tests (ADAS-cog), sMMSE,<br><br>blood tests (CRP, pro-inflammatory cytokine TNF$\alpha$, anti-inflammatory IL10, Anti Pg antibodies) | **Bacterial criteria :** CRP, pro-inflammatory cytokine TNF$\alpha$, anti-inflammatory cytokine IL-10, Ac anti *Pg*<br><br>**Periodontal criteria:** PI, GI, BoP, PPD, number of teeth (Centre for Disease Control/American Academy of Periodontology) | Age, gender, smoking, initial cognitive status |

**Cognitive diseases and diagnosis criteria:** AD, Alzheimer's disease; aMCI, amnestic Mild Cognitive Impairement; NCD, Neurocognitive Disorder; VD, Vascular Disease; ADAS-cog, Alzheimer's Disease Assessment Scale cognitive component; DSM-IV, Diagnostic and statistical Manual of Mental Disorders, 4th version; DSM-V, Diagnostic and statistical Manual of Mental Disorders 5th version; ICD-9, International Statistical Classification of Diseases and Related Health Problems 9th version; ICD-10, International Statistical Classification of Diseases and Related Health Problems 10th version; MMSE, Mini Mental State Examination; MRI Magnetic Resonance Imaging; NINCDS-ADRDA, National Institute of Neurological and Communicative Disorders and Stroke and the Alzheimer's Disease and Related Disorders Association criteria; sMMSE, severe Mini Mental State Examination;

APoE, Apolipoprotein E,e4 allele; hs-CRP, high sensitivity C-reactive protein; IL-10, anti-inflammatory cytokine IL10; TNF $\alpha$, pro-inflammatory cytokine TNF$\alpha$;

**Cofactors:** BMI, Body Mass index;

**Periodontal and oral criteria:** BoP, Bleeding on probing; CAL, Clinical Attachement Loss; CPI, Community Periodontal Index; DMFT, Decayed Missing and Filled Teeth index; PPD, Probing Pocket Deepth; PSR, Probing Screening and Recording index; PI, Plaque Index; GI, gingival index;

**Pathogens:** Aa, Agregatibacter actinomycetemcomitans; An, Actynomyces naeslundii; Co, capnocylophaga ochracea; Cr, Campylobacter rectus; Ec, Eikenella corrodens; En, Eubacterium nodatum; Fn, Fusobacterium nucleatum; Mm; Micromonas micros; Pg, Porhyromonas gingivalis; Pi, Prevotella intermedia; Pm, Prevotella melaninogenica; Pn, Prevotella nigrescens; Si, Streptococcus intermedius; So, Streptococcus oralis; Sm, Streptococcus mutans; Sn, Selenomonas Noxia; Td, Treponema denticola; Tf, Treponema forsythia; Vp, Vellonella parvula;

**Statistics and others:** p, p value; s.d., standard deviation; yo, years old.

**Table S3. Main results of the included studies**

| AUTHOR AND YEAR | MAIN RESULTS | ODDS RATIO | RELATIVE RISK | SIGNIFICANCE |
|---|---|---|---|---|
| **Panzarella et al. 2020, J Alzheimers Dis**<br><br>**(27)** | 1)Patients with AD having a poorer oral health and significantly higher DMFT total score than aMCI (adjusted p = 0.009) and CONS (adjusted p = 0.001)<br><br>2) Significantly different *Fn* load in AD and in CONS (p = 0.04) and after *post-hoc* analysis significantly higher Fn load in AD than in CONS (adjusted p = 0.02) and bacterial load of Td higher in aMCI than in AD (adjusted p = 0.004)<br><br>3)Diagnosis of AD predictive of tooth loss, especially in 80 yo or older subjects | X | X | Periodontal characteristics and AD:<br><br>CPI p = 0.89 n.s.<br><br>PSR p = 0.91 n.s.<br><br>BoP p = 0.11 n.s.<br><br>*Aa* p = 0.39 n.s.<br><br>*Fn* p = 0.04 AD>CONS<br><br>*Pg* p = 0.57 n.s.<br><br>*Pi* p = 0.12 n.s.<br><br>*Td* p = 0.01 aMCI>AD<br><br>*Tf* p = 0.45 |
| **Noble et al. 2014, Plos One**<br><br>**(28)** | 1) Participants with elevated *An* serum IgG (.640 ng/ml) had a higher risk for incident AD, ranging from 80%–100%, with similar yet less precise estimates emerging from more conservative statistical analyses<br><br>remained robust in the fully adjusted Cox model, (age, sociodemographic variables, vascular risk factors, APoE status, stroke history, tobacco abuse, hypertension) (HR)2.0, 95% CI: 1.1–3.8<br><br>2) In a fully adjusted model, high antibody levels to En (.1755 ng/ml) approached statistical significance with a decreased risk of incident AD<br><br>((HR)0.7, 95% CI: 0.4–1.2) | Multivariate analysis:<br><br>*An* Model 4 HR (95% CI) 2.0 (1.1-3.8)<br><br>*En* Model 3 HR (95% CI) 0.7 (0.4-1.2) | X | Periodontal characteristics and AD:<br><br>*An* p = 0.67<br><br>*En* p = 0.47<br><br>*Pg* p = 0.97<br><br>*Tf* p = 0.29<br><br>*Td* p = 0.25<br><br>*Cr* p = 0.33<br><br>*Aa* p = 0.30 |

| | | | | |
|---|---|---|---|---|
| **Syrjälä et al. 2012, Gerontology**<br><br>**(29)** | After adjustment for potential confounding factors, persons with AD and persons with other types of major NCD had an increased likelihood of having teeth with deep periodontal pockets and poor oral hygiene, compared with CONS | Univariate analysis:<br><br>Number of teeth with periodontal pockets ‡4 mm (mean ± SD)<br>Non demented 2.9 (3.8)<br>AD 2.8 (3.3)<br>Multivariate analysis:<br><br>Number of teeth with periodontal pockets (n = 174) – adjusted RR (0.95% CI)<br><br>Non demented 1<br><br>AD 4.7 (3.5-6.3)<br><br>Other types 4.2 (2.4-7.5) | X | X |
| **Beydoun MA. et al. 2020, J Alzheimers Dis**<br><br>**(30)** | -IgG against *Pg, Pm*, Orange-red complex, factor 2 and Si linked to the increase in mortality due to AD in the over 65 yo, this was also true for So in men, while the reverse was true for IgG against *Aa*<br><br>-IgG against *Pg, Cr* and factor 4 linked to an increased risk of AD incidence in people over 65yo, *So* increases the risk of major NCD in men, *Ec* increases the risk of major NCD in women<br>Reverse true for anti *Aa* IgG (over 65 yo), and for *Si* which was marginally and inversely associated with the risk of AD incidence in women<br>-Evidence of an association between periodontal pathogens and AD, stronger for older adults<br><br>-Adds epidemiological evidence suggesting that eradication of *Pg* could | Multivariate analysis:<br><br>Periodontal pathogens and mortality due to AD in the over 65s (Loge (HR) (SE))<br><br>Pg +0.31 (0.11) 0.010<br><br>Pm +0.55 (0.19) 0.005<br><br>Orange-red complex +0.56 (0.17) 0.002<br><br>Periodontal pathogens and risk of AD incidence in people over 65 yo:<br><br>Factor 4 loaded highly on *Cr* and *Pg* titers (aHR=1.22; 95% CI, 1.04-1.43, p=0.012) | X | Periodontal pathogens and mortality due to AD over 65 yo<br><br>*Pg* p = 0.010<br><br>*Pm* p = 0.005<br><br>Red-orange complex p =0.002<br><br>Periodontal pathogens and risk of AD incidence over 65 yo<br><br>Factor 4 (*Pg/Cr*) p =0.012 |

| | | | | |
|---|---|---|---|---|
| | be an effective means of delaying the onset of AD | | | |
| **Ide et al. 2016, Plos One**<br><br>**(31)** | -No clear relation between severity of major NCD and degree of periodontitis, (No subjects with severe major NCD)<br><br>-In AD a bad dental health (periodontitis) is associated with an increase of cognitive decline over a 6 months follow-up period (mean change in the ADAS-cog 2.9 (s.d. 6.6) pts), independently of the basic cognitive state<br><br>-Absence of relationship between low number of teeth (past periodontitis) and cognitive decline: chronic active periodontitis is the most important in the conduct of cognitive decline once AD established<br><br>-No significant relationship between serum levels of anti-Pg antibodies and rates of cognitive decline<br><br>-Evidence of a relative increase in pro-inflammatory status and a decrease in anti-inflammatory status over a 6-month follow-up period in participants with AD and periodontitis<br><br>-If there is a direct relationship between periodontitis and cognitive decline, treatment of periodontitis could be a possible treatment option in AD | Multivariate analysis<br>Mean difference and p value:<br><br>1.Presence or absence of periodontitis at baseline and change in ADAS-cog, points 5.2 (1.7 to 8.8), p = 0.005; *4.9 (1.2 to 8.6), p = 0.01<br><br>2.Presence or absence of periodontitis at baseline and change in sMMSE, points -1.8 (-3.6 to -0.03), p = 0.04; *-1.8 p = 0.06 | X | Multivariate analysis<br>Mean difference and p value:<br><br>1.Presence or absence of periodontitis at baseline and change in ADAS-cog, points 5.2 (1.7 to 8.8), p = 0.005; *4.9 (1.2 to 8.6), p = 0.01<br><br>2.Presence or absence of periodontitis at baseline and change in sMMSE, points -1.8 (-3.6 to -0.03), p = 0.04; *-1.8 p = 0.06 |

**Cognitive diseases and diagnosis criteria:** AD, Alzheimer's disease; aMCI, amnestic Mild Cognitive Impairement; CONS, Controls; ADAS-cog, Alzheimer's Disease Assessment Scale cognitive component; sMMSE, severe Mini Mental State Examination; APoE, Apolipoprotein E;
**Periodontal and oral criteria:** CPI, Community Periodontal Index; BoP, Bleeding on probing; DMFT, Decayed Missing and Filled Teeth index; IgG, Immunoglobulin G; PSR, Probing Screening and Recording index;
**Pathogens:** Aa, Agregatibacter actinomycetemcomitans; An, Actynomyces naeslundii; Cr, Campylobacter rectus; Ec, Eikenella corrodens; En, Eubacterium nodatum; factor 2, *Pi,Pn,Pm*; factor 4, *Pg,Cr*; Fn, Fusobacterium nucleatum; Orange-red complex, *Pm*, *Pi*, *Pn*, *Pg*; Pg, Porhyromonas gingivalis; Pi, Prevotella intermedia; Pm, Prevotella melaninogenica; Pn, Prevotella nigrescens; Si, Streptococcus intermedius; So, Streptococcus oralis; Td, Treponema denticola; Tf, Treponema forsythia;
**Statistics and others:** CI, Confidence Interval; HR, Hazard Ratio; n.s., non significative; p, p value; RR, Relative Risk; SD, Standard Deviation; yo, years old.
